# Piperlongumine is a ligand for the orphan nuclear receptor 4A1 (NR4A1)

**DOI:** 10.3389/fphar.2023.1223153

**Published:** 2023-09-21

**Authors:** Lei Zhang, Greg Martin, Kumaravel Mohankumar, Gus A. Wright, Fuada Mariyam, Stephen Safe

**Affiliations:** ^1^ Department of Veterinary Physiology and Pharmacology, Texas A&M University, College Station, TX, United States; ^2^ Department of Veterinary Pathobiology, Texas A&M University, College Station, TX, United States

**Keywords:** piperlongumine, Nr4a1, cancer, ROS, TXNDC5

## Abstract

Piperlongumine and derivatives are being developed as anticancer agents which act primarily as inducers of reactive oxygen species (ROS) in cancer cell lines. Many of the anticancer activities of piperlongumine resemble those observed for bis-indole derived compounds that bind the orphan nuclear receptor 4A1 (NR4A1) and act as inverse receptor agonists to inhibit NR4A1-regulated pro-oncogenic pathways and genes. In this study we show that like other NR4A1 inverse agonists piperlongumine inhibited RKO, SW480 and HCT116 colon cancer cell growth migration and invasion and induced apoptosis. Piperlongumine also downregulated the pro-reductant isocitrate dehydrogenase 1 (IDH1) and thioredoxin domain-containing 5 (TXNDC5) gene products resulting in the induction of ROS as previously observed for other inverse NR4A1 agonists. ROS also induced sestrin2 and this resulted in activation of AMPK phosphorylation and inhibition of mTOR pathway signaling. It has previously been reported that these pathways/genes are also regulated by inverse NR4A1 agonists or by knockdown of NR4A1. We also observed that piperlongumine directly bound NR4A1, inhibited NR4A1-dependent transactivation and interactions of the NR4A1/Sp1 complex bound to the GC-rich promoter of the NR4A1-regulated G9a gene.

## 1 Introduction

Reactive oxygen species (ROS) and oxidative stress are observed in both non-cancer and cancer cells and are important for maintaining cellular homeostasis and inducing cell death (Ristow et al., 2009; Trachootham et al., 2009; Schumacker Paul, 2015; Chio and Tuveson, 2017). ROS can play a beneficial role in both cancer and non-cancer cells and, drugs that induce ROS are being developed for cancer chemotherapy (Trachootham et al., 2009; Chio and Tuveson, 2017). The cytotoxicity of ROS inducers is associated with their induction of ROS which exceeds the redox buffering capacity of the cell. Some of the commonly used anticancer agents that induce ROS include arsenic trioxide, ionizing radiation, several anthracyclines such as doxorubicin, paclitaxel, and celecoxib (Zhu et al., 2002; Alexandre et al., 2007; Ito et al., 2008; Simůnek et al., 2009; Yoshida et al., 2012; Sritharan and Sivalingam, 2021). For many, of the ROS-inducing anticancer agents their induction of ROS is a contributing factor to their anticancer activity, but it may not be the only factor. For example, doxorubicin not only induces ROS but also intercalates with DNA to inhibit synthesis and also inhibits progression of topoisomerase II (Sritharan and Sivalingam, 2021). A number of natural products and their synthetic analogs that are being developed as anticancer agents also induce ROS in cancer cells and *in vivo* tumor models. These compounds include isothiocyanates, curcumin, piperlongumine, synthetic triterpenoids derived from oleanolic acid and glycyrrhetinic acid, celastrol and many others (Adams et al., 2012; Jutooru et al., 2014; Safe et al., 2018; Kasiappan et al., 2019; Kung et al., 2021; Gabr et al., 2022; Pan et al., 2022; Zhao et al., 2022).

The mechanisms associated with drug-induced ROS are extensive and may be due to targeting mitochondria, inhibition of intracellular pathways/genes associated with redox, depletion of glutathione and other intracellular reductants (Trachootham et al., 2009; Sosa et al., 2013; Castaldo et al., 2016; Chio and Tuveson, 2017; Kim et al., 2019). ROS activates or inactivates genes and pathways that lead to decreased cell growth, induction of apoptosis, inhibition of cell migration, invasion, and metastasis. For example, O’Hagan and coworkers initially reported that treatment of SW480 colon cancer cells with hydrogen peroxide, one form of ROS, induced rapid genome-wide relocation of polycomb members, SIRT1 and DNA methyl transferences from non-GC-rich to GC-rich promoter sequences (O’Hagan et al., 2011). The resulting modulation of gene expression included downregulation of cMyc, and subsequent studies show that this can lead to decreased cell growth, survival and migration/invasion (Safe et al., 2018).

Studies in this laboratory have identified a series of bis-indole derived (CDIM) compounds that bind to the orphan nuclear receptor 4A1 (NR4A1) and act as inverse agonists to inhibit multiple pro-oncogenic NR4A1-dependent genes/pathways associated with cancer cell growth, survival and migration/invasion (Safe et al., 2021; Safe and Karki, 2021). In some cell lines the CDIM compounds downregulate the pro-reductant thioredoxin domain containing 5 (TXNDC5) and isocitrate dehydrogenase 1 (IDH1) genes and this is accompanied by induction of ROS (Lee et al., 2014a; Lee et al., 2014b; Hedrick et al., 2015; Lacey et al., 2016; Mohankumar et al., 2019). Moreover, knockdown of NR4A1 or TXNDC5 by RNA interference also induced ROS in cancer cell lines suggesting that some ROS-inducing anticancer agents may also be NR4A1 ligands and that their anticancer activities may due, in part, to their activity as inverse NR4A1 agonists in cancer cells. This is consistent with results of studies with the potent ROS-inducing anticancer agent celastrol (Zhao et al., 2022) which has now also been identified as an NR4A1 ligand (Hu et al., 2017). This observation is relevant with respect to drug development since NR4A1 has been implicated as a potential drug target for multiple diseases including cancer and many other inflammatory diseases (Pearen and Muscat, 2010; Kurakula et al., 2014; Zhang et al., 2018; Chen et al., 2020). In this study we have investigated the anticancer activity of piperlongumine, a well-known ROS-inducing anticancer agent (Kung et al., 2021; Pan et al., 2022) and show for the first time that this compound is an NR4A1 ligand acting as an inverse agonist in colon cancer cells.

## 2 Methods

### 2.1 Cell culture, reagents, and antibodies

RKO, SW480 and HCT116 (RRID: CVCL_0291) colon cancer cells are purchased from American Type Culture Collection (Manassas, VA) and validation on selected cell lines were determined by biosynthesis (Lewisville, TX). Cells are cultured in DMEM medium with 10% FBS at 37 °C in the presence of 5% CO2. The details of antibody using for Western blotting and ChIP assays are shown in Supplementary Table S1.1, 1-Bis(3′-indolyl)-1-(3,5-dichlorophenyl)methane (DIM-3,5-CI2) was synthesized by coupling indole and 3,5-dichlorobenzaldehyde as described (Lee et al., 2014a) and piperlongumine was purchased from MilliporeSigma (Burlington, MA).

### 2.2 Direct binding assay

The recombinant LBD of NR4A1 (0.5 μmol/L) in 1.0 mL of phosphate buffered saline (pH 7.4) was incubated for 3 min at 25 °C in a temperature-controlled fluorescence spectrometer (Varian Cary Eclipse). Fluorescence was measured using an excitation wavelength of 285 nm (excitation slit width = 5 nm) and an emission wavelength ranging from 300 to 420 nm (emission slit width = 5 nm). Aliquots of piperlongumine in DMSO were added, incubated at 25 °C for 3 min. The loss of fluorescence curve was measured, and K_D_ values were determined from the net fluorescence curve (background ligand fluorescence is subtracted). The fluorescence spectrum curve was derived using the internal filter correction and subtractions of the background fluorescence of the compound alone.

### 2.3 Isothermal titration calorimetry

Isothermal titration calorimetry (ITC) was used to determine the ligand binding constant (K_D_) to NR4A1 utilizing an Affinity ITC (TA Instruments, New Castle, DE). Briefly, the experimental setup was as follows. The ITC sample cell contained 250 μL of NR4A1 protein (ligand binding domain, LBD) at a concentration of 20 μmol/L in buffer containing 20 mmol sodium phosphate/L (pH 7.4), 5% glycerol, and 1.0% ethanol. The ligand titrant was prepared in the same buffer as above at a ligand concentration of 100 μmol/L. The initial ligand stock solution was prepared at a final concentration of 20 mmol ligand/L DMSO prior to preparation of the ligand titrant. The ligand titration into protein was performed at 25 °C with a stir rate of 125 rpm. Each ligand injection volume was 2.5 μL followed by up to 300 s to measure the total heat flow required to maintain constant temperature. A total of thirty injections were performed for each ligand/NR4A1 combination. In a separate set of injections, the same ligand was injected into buffer only (no protein) in order to determine heat flow as a result of ligand dilution into buffer. The ligand/buffer values were subtracted from the ligand/protein values prior to data analysis using the Affinity ITC manufacturer-supplied data analysis software package. The resulting data are plotted as heat flow (μJ) *versus* the molar ratio of injected ligand to NR4A1 in the sample cell.

### 2.4 Computation-based molecular modeling

Molecular modeling studies were conducted using Maestro (Schrödinger Release 2020–1, Schrödinger, LLC, New York, NY, 2020). The version of Maestro used for these studies is licensed to the Laboratory for Molecular Simulation (LMS), a Texas A&M University core user facility for molecular modeling and is associated with the Texas A&M University High Performance Research Computing (HPRC) facility (College Station, TX, 77,843). All Maestro-associated applications were accessed via the graphical user interface (GUI) VNC interactive application through the HPRC Ada OnDemand portal. The crystal structure coordinates for human orphan nuclear receptor NR4A1 ligand binding domain (LBD) (Zhan et al., 2012) were downloaded from the Protein Data Bank (https://www.rcsb.org; PDB ID 3V3Q). The human NR4A1 LBD crystal structure was prepared for ligand docking utilizing the Maestro Protein Preparation Wizard; restrained minimization of the protein structure was performed utilizing the OPLS3e force field. Each ligand (piperlongumine or DIM-3,5-Cl2) three-dimensional structure was prepared for docking utilizing the Maestro LigPrep, again using the OPLS3e force field. Maestro Glide (Friesner et al., 2004; Halgren et al., 2004; Friesner et al., 2006) was utilized with the default settings to dock each prepared ligand to the prepared protein, predict the lowest energy ligand binding orientation, and calculate the predicted binding energy in units of kcal/mol.

### 2.5 Cell proliferation assay

Cell proliferation was investigated using XTT Cell Viability Kit (Cell Signaling Biotechnology) according to the manufacturer’s instructions. Cells (1.5 × 10^4^/well) were plated in 100 μL of plating medium (as above) in 96-well plates and allowed to attach for 24 h. The medium was then changed to DMEM containing 2.5% charcoal-stripped FBS, and either vehicle (dimethyl sulfoxide (DMSO)) or designed concentrations of compounds in DMSO were added. After 24 and 48 h of culture, 35 μL of XTT reaction solution (sodium 3′-[1-(phenyl-aminocarbonyl)-3,4-tetrazolium]-bis(4-methoxy-6-nitro) benzenesulfonic acid hydrate and N-methyl dibenzopyrazine methyl sulfate; mixed in proportion 50:1) was added to the each well. The optical density was read at 450 nm wavelength in a plate reader after 4 h of incubation. All determinations were replicated in at least three separate experiments.

### 2.6 Transfection, luciferase assay, and rescue assay

Cells were plated on 12-well plates at 5 × 10^4^/well in DMEM medium supplemented with 2.5% charcoal-stripped FBS. After 24 h growth, various amounts of plasmid DNA [i.e., UASx5-Luc (400 ng), GAL4-NR4A1 (250 ng) and β-gal (250 ng)] were cotransfected into each well by GeneJuice Transfection reagent (Millipore Sigma, Darmstadt, Germany) according to the manufacturer’s protocol. After 6 h of transfection, cells were treated with plating media (as indicated above) containing either solvent (DMSO) or the indicated concentration of compound (in DMSO) for 18 h. Cells were then lysed using a freeze–thaw protocol and 30 μL of cell extract was used for luciferase and β-gal assays. LumiCount (Packard, Meriden, CT) was used to quantify luciferase and β-gal activities. Luciferase activity values were normalized against corresponding β-gal activity values as well as protein concentrations determined by Bradford assay. The rescue assay used FLAG-NR4A1 expression vector to partially inhibit downregulation of TXNDC5 by piperlongumine.

### 2.7 Tunel staining assay

Cell death was assessed using terminal deoxynucleotidyl transferase dUTP nick end labeling (TUNEL). In brief, 5.0 × 10^4^ cells were seeded on 24-well plates that attached with glass coverslips and allowed 24 h to attach. After treating with the compound for 24 h, cells were fixed in 4% paraformaldehyde and then stained using the TUNEL kit (Elabscience, Wuhan, China) for 3 h at 37 °C. Cell nuclei were counterstained with DAPI for 5 min. Cells were imaged using ImageXpress Confocal HT. ai High-Content Imaging System (Molecular Devices, San Jose, CA).

### 2.8 Boyden Chamber invasion assay and scratch migration assay

Attached cells (2.0 × 10^5^) were treated with DMSO or with different concentration of piperlongumine in DMEM medium supplemented with 2.5% charcoal stripped FBS for 24 h. For Boyden chamber invasion assay, 1.0 × 10^5^ cells from each treatment condition were allowed to invade through the Boyden Chamber for 48 h. Cells that invaded into the Boyden Chamber were fixed using formaldehyde, stained, and then counted. For the scratch migration assay, cells were grown to 90% confluency in 6-well plates then scratched with a 200 μL sterile pipette tip and washed with PBS to remove detached cells from the plates. Cells were maintained in an incubator with DMSO or indicated treatments for 48 h and cells were then fixed with 4% formaldehyde and stained with crystal violate solution. The wound gap was observed under AMG EVOS fl microscope. At least 3 replicates were performed for each treatment group.

### 2.9 Western blot analysis

Cells (3.0 × 10^5^) were seeded on 6-well plate and after various treatments, whole cell lysates were obtained by treating them with high salt lysis buffer RIPA (Thermo Scientific, Waltham, MA) that contained protease and phosphatase inhibitors (GenDEPOT, Baker, TX). The total protein in the lysates was quantified by Bradford assay. Equal amounts of protein from each lysate were then loaded on SDS polyacrylamide gel; 35 µg of whole cell lysate were run in 12% of SDS page gels for survivin and c-caspase-3 proteins; 35 µg of whole cell lysate were run in 8% of SDS page gels for G9a, mTOR and p-mTOR proteins and 25 µg of whole cell lysate were run in 10% of SDS page gels for the remaining proteins. The proteins from the gel were transferred to a PVDF membrane, then blocked for 1 hour using 5% skimmed milk. The membranes were incubated with primary antibody for 12 h at 4 °C, then washed with Tris-buffered saline and Polysorbate 20 (TBST) and incubated with HRP-linked secondary antibody for 1 h at 20 °C. The membranes were further washed with TBST and treated with Immobilon western chemiluminescence HRP-substrates to detect the protein bands using Kodak 4000 MM Pro image station (Molecular Bioimaging, Bend, OR, United States).

### 2.10 ChIP assay

The chromatin immunoprecipitation (ChIP) assay was performed using the ChIP-IT Express magnetic chromatin immunoprecipitation kit (Active Motif, Carlsbad, CA) according to the manufacturer’s protocol. Cells (3 × 10^7^) were treated with DMSO or indicated concertation of piperlongumine for 24 h. Cells were then fixed with 1% formaldehyde, and the cross-linking reaction was stopped by addition of 0.125 M glycine. After washing twice with phosphate-buffered saline, cells were scraped and pelleted. Collected cells were hypotonically lysed, and nuclei were collected. Nuclei were then sonicated to the desired chromatin length (200–1,500 bp). The sonicated chromatin was immunoprecipitated with 3 µg of normal IgG (abcam), NR4A1 (Abcam), Sp1 (Abcam), or RNA polymerase II (pol II; Abcam) antibodies and protein G-conjugated magnetic beads at 4 °C for overnight. After the magnetic beads were extensively washed, protein-DNA cross-links were reversed and eluted. Reversed cross-link DNA was prepared by proteinase K digestion followed by Chromatin IP DNA purification (Active Motif). Purified DNA products were then analyzed by quantitative real-time PCR using amfiSure qGreen Q-PCR master mix (genDEPOT) using the manufacturer’s protocol. The primers for detection of the G9a promoter region were F: 5′- CAG​ATG​GGG​ACA​GAG​ACG​C -3′, R: 5′- CCCGGAGCATTGCACG -3’.

### 2.11 Statistical analysis

Each assay was performed in triplicate and the results were presented as means with standard deviation (SD). The statistical significance of differences between the treatment groups was determined by Dunnett’s multiple comparison test in ordinary one-way ANOVA. Gel analysis of Western blotting was done using ImageJ (1.53K) software (RRID:SCR_003070). GraphPad Prism 8 (Version 8.4.3) software (RRID:SCR_002798) was used for analysis of variance and determine statistical significance. Data with a *p*-value of less than 0.05 were considered statistically significant and indicated (*) in the figures.

## 3 Results

### 3.1 Piperlongumine binds the orphan nuclear receptor 4A1 (NR4A1) and inactivated NR4A1-dependent transcription

**FIGURE 1:**
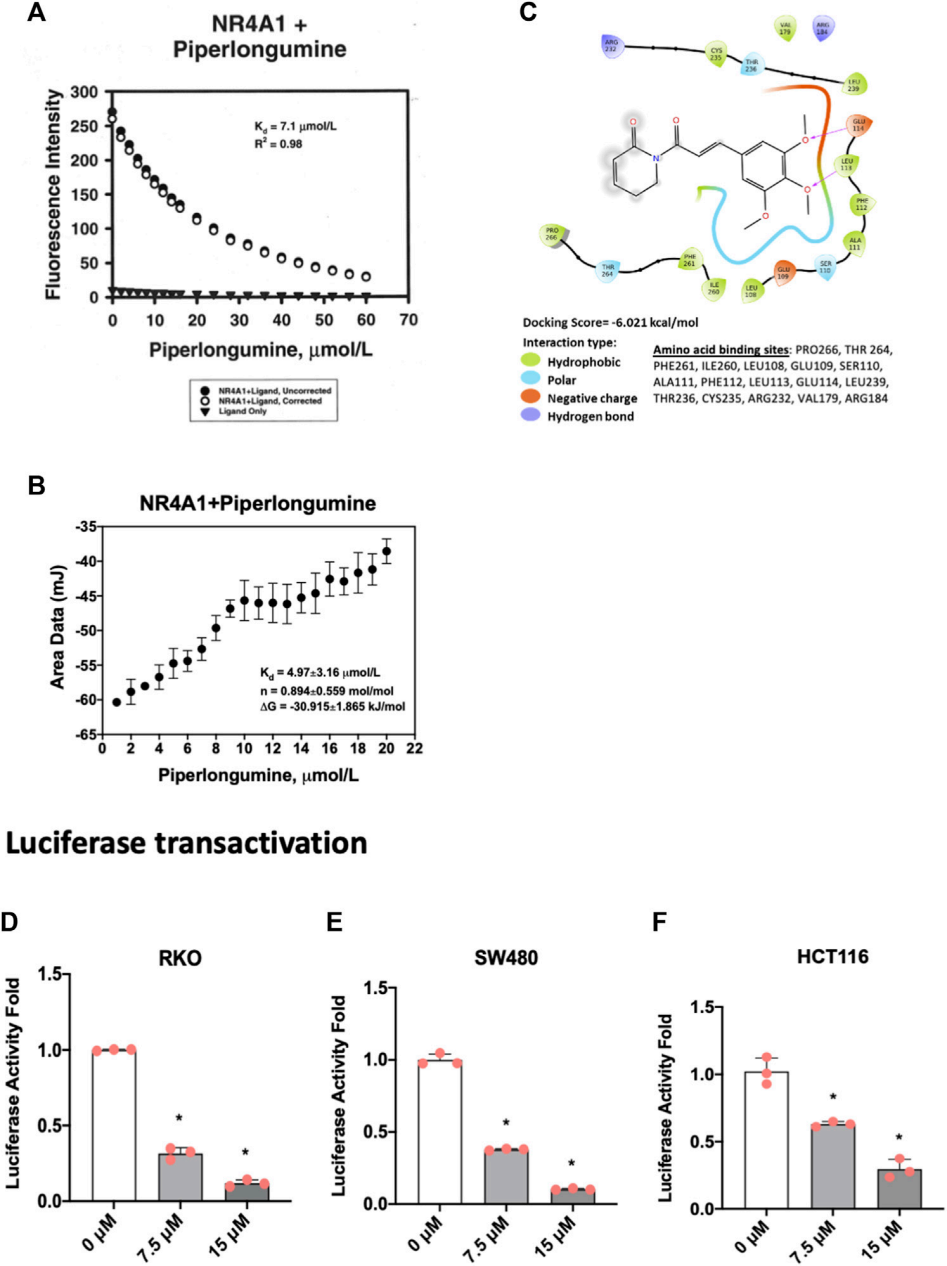
Piperlongumine as an NR4A1 ligand. **(A)** Direct binding of piperlongumine to the LBD of NR4A1 by measuring loss of Trp fluorescence as outlined in the Methods; **(B)** Binding of piperlongumine to the LBD of NR4A1 was also determined using an ITC assay as outlined in the Methods; **(C)** Piperlongumine interactions with LBD of NR4A1 were modeled using Schrodinger/Maestro as outlined in the Methods. Effects of piperlongumine on NR4A1-dependent transactivation were determined in RKO **(D)**, SW480 **(E)** and HCT116 **(F)** colon cancer cells transfected with a GAL4-NR4A1 chimera and a UAS-luc reporter gene as outlined in the Methods. Results **(D–F)** are expressed as means ± SD for at least 3 determinations and significant (*p* < 0.05) effects of piperlongumine compared to control (DMSO) are indicated (*).

Based on the functional similarities between ROS-inducing anticancer agents and NR4A1 inverse agonists in cancer cell lines we initiated studies on the potential activity of piperlongumine as an NR4A1 ligand. Figure 1A illustrates the direct binding of piperlongumine to the ligand binding domain (LBD) of NR4A1 using an assay which measures the loss of fluorescence of a Trp residue in the LBD (Lee et al., 2014a). The results show that piperlongumine binds NR4A1 with a K_D_ value of 7.1 µM and as a positive control for this assay we observed a similar binding curve for the known NR4A1 ligand celastrol with a K_D_ value of 2.3 µM (data not shown). Figure 1B illustrates the interaction of piperlongumine and NR4A1 as determined in the ITC assay and the K_D_ and ΔG values were 4.97 μmol/L and −30.9 kj/mol respectively. The lower K_D_ value in the ITC assay may represent interactions of piperlongumine not only within the binding pocket but also other sites in the LBD or NR4A1.

Figure 1C illustrates Maestro/Schrodinger modeling of piperlongumine with the LBD of NR4A1 and shows interactions of piperlongumine with hydrophobic (Pro266, Thr264, Phe261, Leu108, Glu109, Ser110), polar (Ala111, Phe112, Leu113, Glu114, Leu239), and negatively charged (Thr236, Cys235, Arg232, Val179 and Arg184) amino acids. These results (Figures 1A–C) clearly demonstrate that piperlongumine is an NR4A1 ligand and this is further confirmed in NR4A1-dependent transactivation studies in cells transfected with a GAL4-NR4A1 chimera and a GAL4-dependent reporter gene (UAS-luc). The results show that piperlongumine decreased luciferase activity in RKO (Figure 1D), SW480 (Figure 1E), and HCT116 (Figure 1F) colon cancer cells demonstrating the NR4A1 inverse agonist activity of piperlongumine for this transactivation response.

### 3.2 Piperlongumine inhibited colon cancer cell growth and induces apoptosis

**FIGURE 2:**
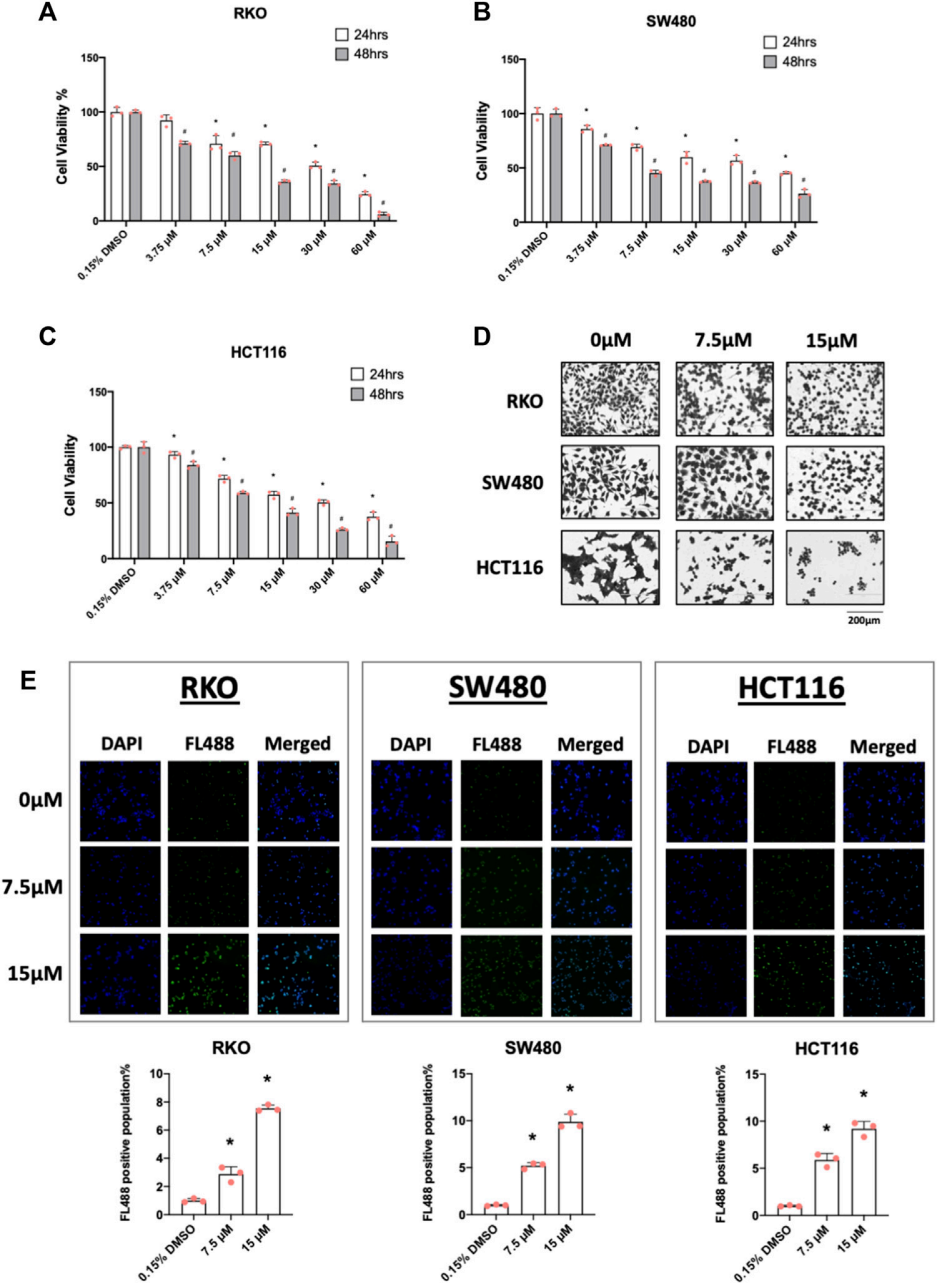
Piperlongumine inhibits colon cancer cell growth and induces cell death in a TUNEL staining assay. RKO **(A)**, SW480 **(B)** and HCT116 **(C)** cells were treated with different concentrations of piperlongumine for 24 or 48 h and effects were measured using XTT assay as outlined in the Methods. **(D)** Colon cancer cells were treated for 24 h with 7.5 or 15 µM piperlongumine and changes in cell morphology were determined as outlined in the Methods. **(E)** Colon cancer cells were treated for 24 h with piperlongumine and the TUNEL assay was used to determine cell death as outlined in the Methods. Results are expressed as means ± SD for at least 3 determinations and significant (*p* < 0.05) induction is indicated (*).

In cancer cells inverse NR4A1 agonists decrease cancer cell growth, induce apoptosis and inhibit migration/invasion (Lee et al., 2014a; Safe et al., 2021) and treatment of RKO, SW480 and HCT116 cells with 7.5 and 15 µM piperlongumine inhibited cell growth (Figures 2A–C) as determined in an XTT assay. Treatment with 7.5 and 15 µM piperlongumine caused the morphology of colon cancer cells to visibly shrink and change into a rounded shape (Figure 2D). Moreover, this same treatment protocol was also used to show that piperlongumine induced apoptosis using the TUNEL assay (Figure 2E).

### 3.3 Piperlongumine inhibited colon cancer cell growth, migration and invasion

**FIGURE 3:**
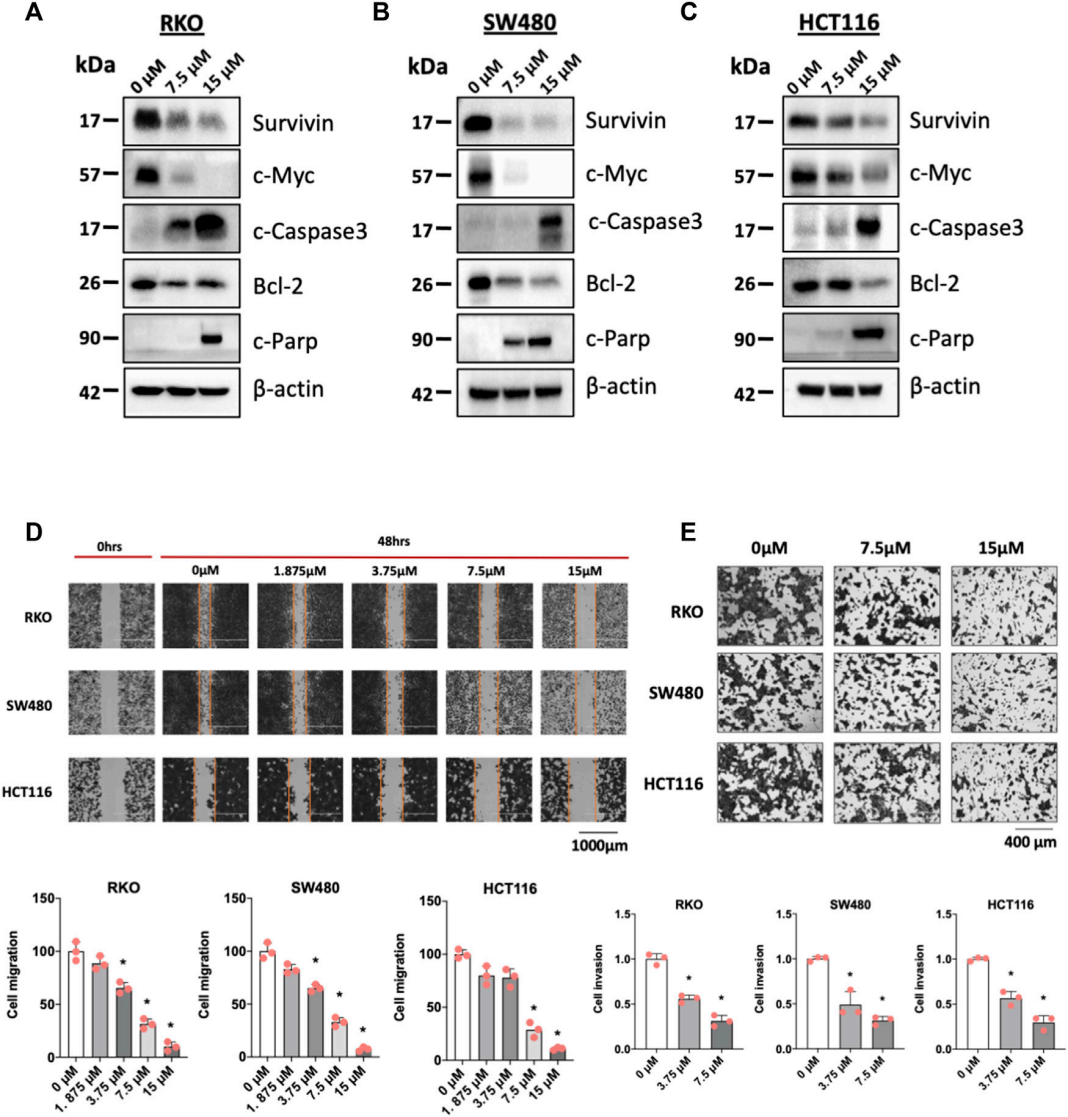
Piperlongumine induces apoptosis, inhibits migration and invasion in colon cancer cell lines. RKO **(A)**, SW480 **(B)** and HCT116 **(C)** colon cancer cells were treated with 7.5 or 15 µM piperlongumine for 24 h and whole cell lysates were obtained and analyzed by western blots as outlined in the Methods and bands were quantitated relative to β-actin and intensities are given as means ± SD for at least 3 determinations and significant effects (*p* < 0.05) relative to the control (DMSO) treatment groups are indicated (*). Colon cancer cells were treated with piperlongumine for 24 h and effects on cell migration and cell invasion **(D and E)** were determined in scratch and Boyden chamber assays respectively as outlined in the Methods. Quantitative results are expressed as means ± SD for at least 3 determinations and significant (*p* < 0.05) differences between control (DMSO) and piperlongumine treated cells is indicated (*).

Figure 3 illustrates that after treatment with piperlongumine for 24 h there was a decrease in the growth-promoting oncogene cMyc and several markers of apoptosis, namely, decreased bcl-2 and survivin and increased cleaved caspase 3 and PARP in the 3 colon cancer cell lines (Figures 3A–C). Figure 3D illustrates and quantitates the effects of piperlongumine on migration of RKO, SW480 and HCT116 colon cancer cell lines. A time 0 the migration lane was created and after 48 h there was significant migration of cells into this lane and the relative amount of migrated cells was set at 100%. Treatment of cells with 1.875—15 µM significantly inhibited cell migration after 48 h, however, at the higher doses this was also due, in part, to the cytotoxicity of piperlongumine. In contrast, at the low concentrations of piperlongumine (1.875 and 3.75 µM) cell migration was still significantly inhibited in RKO and SW480 cells and cytotoxicity was minimal. In HCT116 cells there was also some inhibition of migration at the two lower doses, but it was not significant. Results of a Boyden chamber assay on cell invasion showed that 3.75 and 7.5 µM piperlongumine also inhibited invasion of colon cancer cells (Figure 3E). These results (Figures 3D,E) demonstrate that piperlongumine affects functional responses in colon cancer cells that are consistent with their activity as inverse NR4A1 agonists and effects of NR4A1 knockdown (Safe et al., 2021). We also show that in contrast with results observed for bis-indole derived inverse NR4A1 agonists and resveratrol that piperlongumine did not affect expression of β1-integrin and other integrins (Supplementary Figure S1D) (Zhang et al., 2022).

### 3.4 Piperlongumine downregulated pro-reductant genes, induces ρAMPK and sestrin2

**FIGURE 4:**
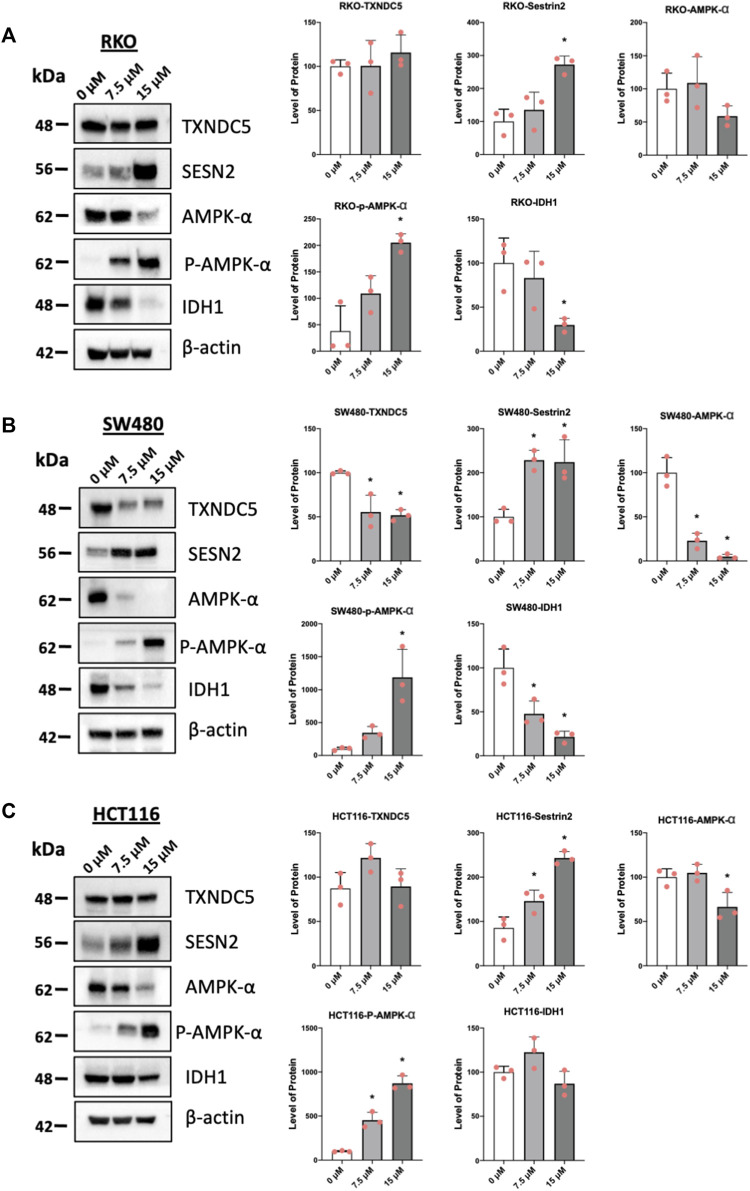
Piperlongumine affects redox in colon cancer cell lines. RKO **(A)**, SW480 **(B)** and HCT116 **(C)** colon cancer cells were treated with 7.5 or 15 µM piperlongumine for 24 h and whole cell lysates were obtained and analyzed by western blots as outlined in the Methods and bands were quantitated relative to β-actin. Band intensities are given as means ± SD for at least 3 determinations and significant effects (*p* < 0.05) relative to the control (DMSO) treatment groups are indicated (*).

One of the hallmarks of NR4A1 inverse agonists is the downregulation of pro-reductant genes such as TXNDC5 and IDH-1 which results in increased reactive oxygen species and induction of the oxygen sensor sestrin2 (SESN2) and SESN 2-dependent activation of AMPK (Lee et al., 2014a; Lee et al., 2014b; Hedrick et al., 2015; Lacey et al., 2016; Mohankumar et al., 2019; Shrestha et al., 2020). Treatment of the colon cancer cells with piperlongumine resulted in downregulation of IDH1 in RKO (Figure 4A), SW480 (Figure 4B), and HCT116 (not significant) (Figure 4C) cells and selective downregulation of TXNDC5 in only SW480 cells. Supplementary Figure S1E illustrates that overexpression of FLAG-NR4A1 partially rescues the effects of piperlongumine downregulation of TXNDC5 in SW480 cells. Higher levels of FLAG-NR4A1 were less effective. Induction of sestrin2 and activation (increased phosphorylation) of AMPK was observed in all 3 cell lines. NR4A1 regulates multiple reductant genes (Lee et al., 2014b) and their selective regulation by bis-indole-derived CDIMs and piperlongumine is consistent with their activity as selective NR4A1 modulators.

### 3.5 Piperlongumine induced ROS and ROS-dependent sestrin2 in colon cancer cells and inhibited mTOR

**FIGURE 5:**
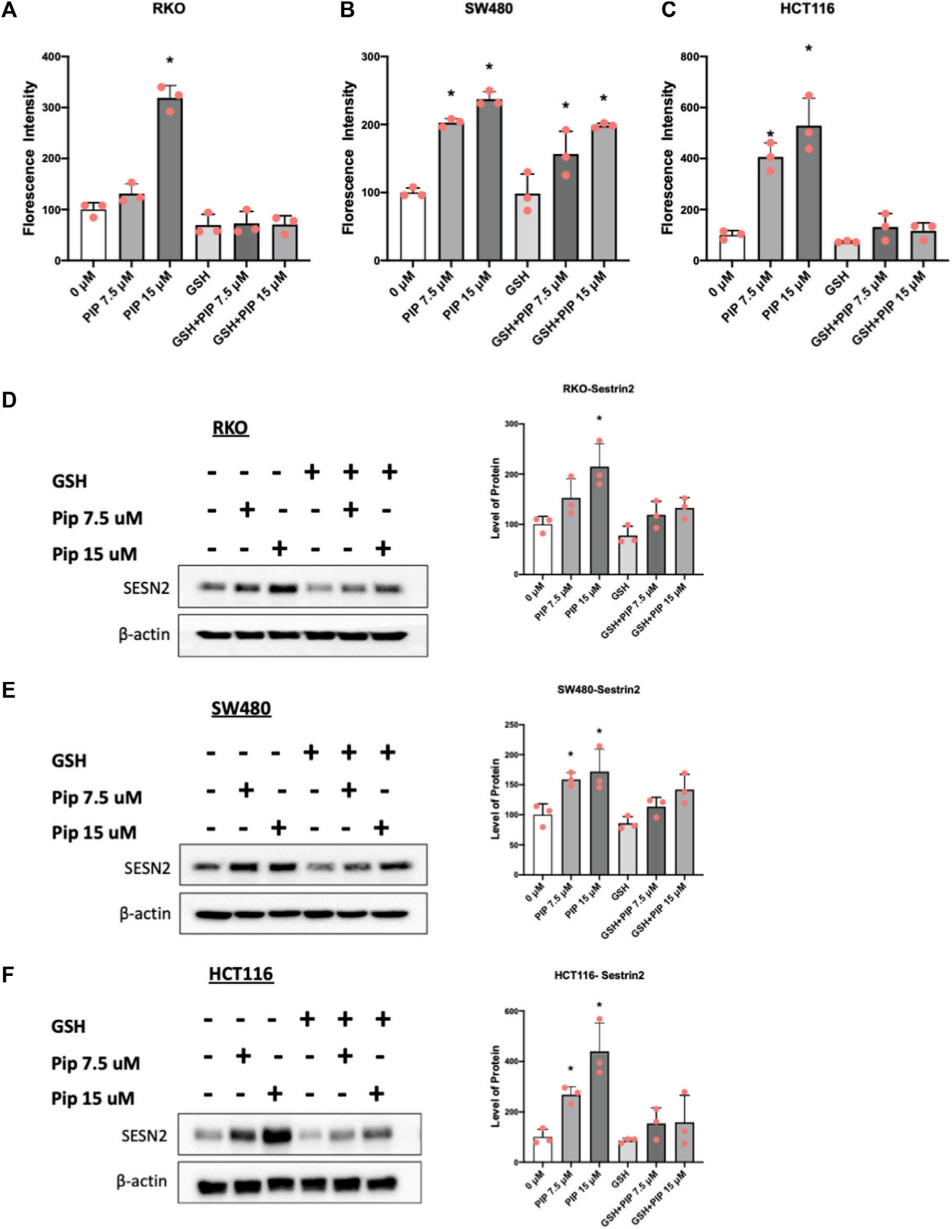
Piperlongumine induces ROS and sestrin2. Treatment of RKO **(A)**, SW480 **(B)** and HCT116 **(C)** cells with piperlongumine, 5 ϻM glutathione and a combination of piperlongumine plus glutathione. Piperlongumine increased ROS induction due to metabolism of cell permeable DCFDA as outlined in the Methods. The same treatment protocol was used in RKO **(D)**, SW480 **(E)** and HCT116 **(F)** cells and whole cell lysates were analyzed by western blots and band intensifies were quantitated relative to β-actin. Results are expressed as means ± SD for at least 3 determinations and significant effects (*p* < 0.05) compared to control are indicated (*) and attenuation by glutathione is also indicated (*).

**FIGURE 6:**
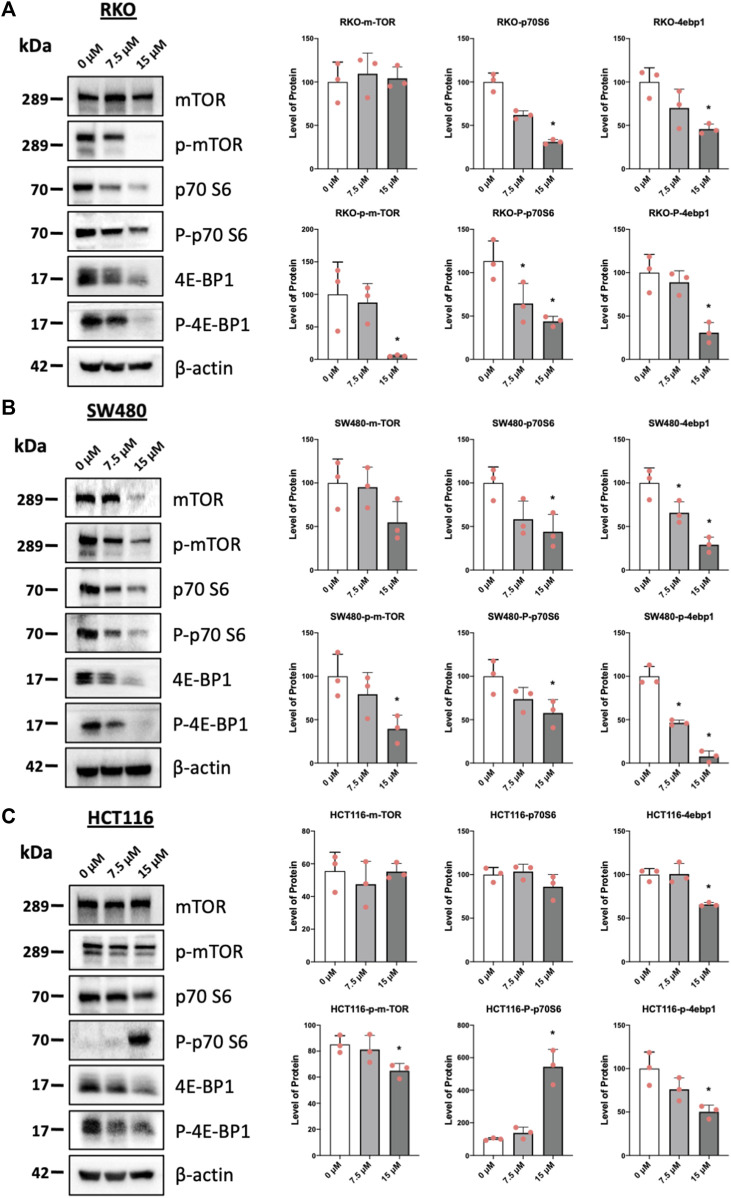
Piperlongumine inhibits mTOR in colon cancer cells. RKO **(A)**, SW480 **(B)** and HCT116 **(C)** cells were treated with piperlongumine for 24 h and whole cell lysates were obtained and analyzed by western blots and band intensities were quantitated relative to β-actin. Band intensities are given as means ± SD for at least 3 determination and significant changes (*p* < 0.05) compared to control (DMSO) are indicated (*).

Results illustrated in Figure 5 summarize effects of piperlongumine alone on induction of ROS and sestrin2 and effects of piperlongumine in combination with the antioxidant glutathione. Treatment of colon cancer cells with piperlongumine increases ROS-dependent fluorescence in all 3 cell which is clue to oxidation of the cell permeant DCFDA into its fluorescent metabolite (Figures 5A–C). In addition, this was accompanied by induction sestrin2 (Figures 5D–F) and the magnitude of the fluorescent and sestrin2 induction responses were decreased after cotreatment with glutathione. Sestrin2-dependent activation of AMPKα inhibits mTOR signaling and results in Figures 6A–C show that piperlongumine significantly decreased levels of phosphor-mTOR in the 3 colon cancer cell lines. Moreover, inhibition of mTOR is accompanied by significantly decreased phosphorylation of 4 E-BP1 and p70S6 in the 3 colon cancer cell lines.

### 3.6 Piperlongumine downregulated G9a levels and decreases association of NR4A1 and Sp1 with the G9a promoter

**FIGURE 7:**
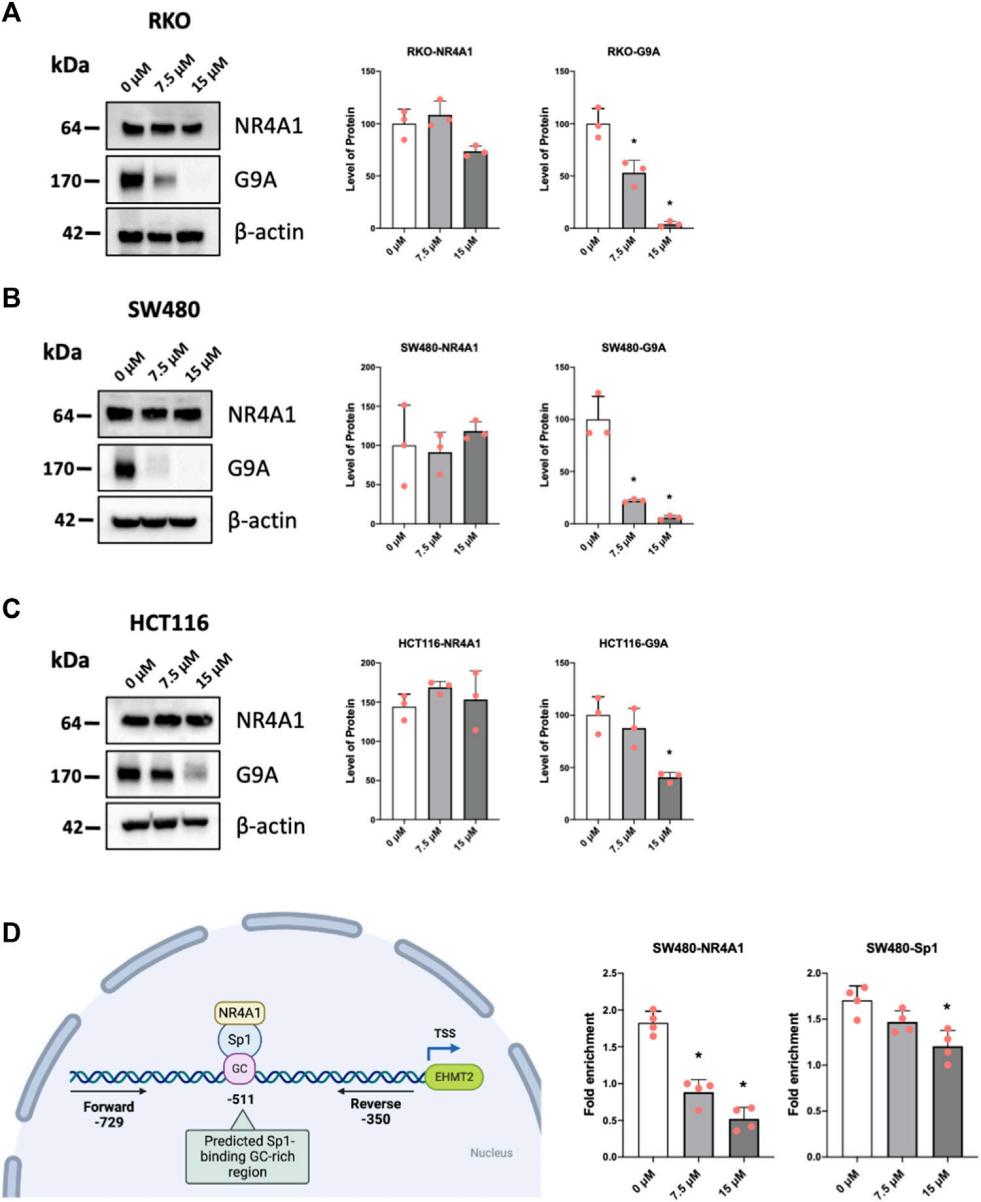
Mechanism of G9a regulation by piperlongumine. RKO **(A)**, SW480 **(B)** and HCT116 **(C)** colon cancer cells were treated with 7.5 or 15 µM piperlongumine for 24 h and whole cell lysates were obtained and analyzed by western blots as outlined in the Methods and bands were quantitated relative to β-actin. **(D)** A ChIP assay was used to determine the interactions of NR4A1 and Sp1 with the GC-rich region of the G9a gene promoter as outlined in the Methods using primers that encompass the GC-rich region. QPCR was used to analyze fold enrichment of NR4A1 and Sp1 associated with the GC-rich promoter region and QPCR intensities are given as means ± SD for at least 3 determinations and significant effects (*p* < 0.05) relative to the control (DMSO) treatment groups are indicated (*).

Previous studies have demonstrated that the histone methyltransferase G9a gene is regulated by NR4A1/Sp1 where NR4A1 acts as a ligand-dependent cofactor of Sp1 which in turn is bound to the GC-rich promoter of the G9a gene (Shrestha et al., 2020). Results in Figures 7A–C show that piperlongumine downregulates G9a expression in RKO, SW480 and HCT116 cells and this parallels effects observed for bis-indole derived CDIM compound, quercetin and kaempferol which also bind NR4A1 and act as inverse NR4A1 agonists (Shrestha et al., 2020; Shrestha et al., 2021). Chromatin immunoprecipitation and QPCR were used to quantitatively detect interactions of NR4A1 and Sp1 with the GC-rich G9a promoter (Figure 7D) in SW480 cells. Treatment with piperlongumine had minimal effects on Sp1 binding but significantly decreased NR4A1 interactions in this region of the G9a promoter and similar results were previously observed using bis-indole derived NR4A1 ligands in Rh30 cells (Shrestha et al., 2020). This data is consistent with interaction of piperlongumine and NR4A1 with DNA bound Sp1 on the G9a promoter as previously observed for other inverse NR4A1 agonists that inhibit NR4A1/Sp-regulated gene expression (Lee et al., 2010; Hedrick et al., 2016; Shrestha et al., 2020).

## 4 Discussion

The orphan nuclear receptor NR4A1 is an immediate early gene that plays an important role in maintaining cellular homeostasis and in pathophysiology (Pearen and Muscat, 2010; Kurakula et al., 2014; Zhang et al., 2018; Chen et al., 2020). For example, NR4A1 is elevated by stressors and inflammatory agents and levels are increased in many solid tumors, fibrosis, some cardiovascular, neuronal and metabolic diseases (Pearen and Muscat, 2010; Kurakula et al., 2014; Zhang et al., 2018; Chen et al., 2020). NR4A1 ligands such as cytosporone B act as disease-specific agonists or inverse agonists for relieving symptoms of these diseases including cancer (Liu et al., 2010; Zhan et al., 2012). Studies in this laboratory have characterized the inverse agonist activities of bis-indole derived NR4A1 ligands in solid tumor-derived cancers and their beneficial effects in neuronal disease, endometriosis, and glucose uptake into muscle cells has also been reported (Lee et al., 2014a; Lee et al., 2014b; Hammond et al., 2015; Hedrick et al., 2015; Lacey et al., 2016; Mohankumar et al., 2018; Mohankumar et al., 2019; Chatterjee et al., 2020; Mohankumar et al., 2020; Shrestha et al., 2020; Karki et al., 2021; Safe et al., 2021; Safe and Karki, 2021; Shrestha et al., 2021). Many therapeutic agents including some health-promoting natural products exhibit multiple activities and this can lead to drug repurposing which allows a particular drug to be used for more than one mechanism-based response (Pushpakom et al., 2019).

Natural products including several anticancer agents induce responses similar to that observed for bis-indole derived NR4A1 ligands which act as inverse agonists and inhibit NR4A1-dependent pro-oncogenic genes and pathways including cell growth, survival, and related genes (Safe and Karki, 2021). It was previously reported that the flavonoid kaempferol downregulated expression of G9a in gastric cancer cells (Kim et al., 2018) and studies in this laboratory reported that the flavonoids quercetin and kaempferol bind NR4A1 and downregulate G9a and other NR4A1-regulated genes in Rh30 cells (Shrestha et al., 2021) and similar results have now been observed for piperlongumine (Figure 7). Several other natural products that act as anticancer agents including cytosporone B, celastrol, resveratrol and some alkaloids have also been identified as NR4A1 ligands and this will help facilitate their repurposing for treating diseases such as cancer and other inflammatory diseases in patients that highly express this receptor (Liu et al., 2010; Hu et al., 2017; Safe et al., 2021; Shrestha et al., 2021; Zhang et al., 2022).

Piperlongumine contains two α,β-unsaturated ketone moieties and previous studies show that piperlongumine-protein adduction via the Michael reaction occurs primarily at the two to three double bond, and this can contribute to induction of ROS in some cancer cell lines (Adams et al., 2012). Previous studies have demonstrated that piperlongumine forms covalent adducts with proteins and in this study, we assume that some of the activity of piperlongumine may be due, in part to the covalent adduct of NR4A1. In this study we observed a pattern of ROS induction and inhibition of mTOR which is consistent with the binding of piperlongumine to NR4A1 and its activity as an inverse NR4A1 agonist. The effects of other inverse NR4A1 agonists or NR4A1 knockdown include downregulation of one or both productant genes IDH1 and TXNDC5, induction of ROS, ROS-dependent activation of sestrin2, sestrin2-dependent activation of AMPK which in turn inhibits mTOR activation (Lee et al., 2014a; Lee et al., 2014b; Hedrick et al., 2015; Lacey et al., 2016; Mohankumar et al., 2019). This pathway was also observed in colon cancer cells treated with piperlongumine (Figure 4, Figure 5, Figure 6) and the downregulation of the NR4A1-regulated pro-reductant genes has not previously been observed in studies with piperlongumine. It is unlikely that these receptor-mediated responses are due to an alkylated piperlongumine-receptor complex and accompanying conformational changes however, these results do not preclude the contribution of piperlongumine adducts to the observed induction of ROS and downstream genes/pathways. It should also be noted that there were colon cancer cell context-dependent differences in the effects of piperlongumine on downregulation of IDH-1 and TXNDC5 (Figure 4). We also observed that piperlongumine did not affect expression of other genes such as β-1 and other integrins which are downregulated by bis-indole derived NR4A1 inverse agonists (Safe and Karki, 2021). This selectivity of piperlongumine and other compounds that bind NR4A1 may be due to their activity as selective NR4A1 ligands which has previously been observed for many other nuclear receptors (Burris et al., 2013). The selectivity of receptor ligands is associated with multiple factors including their ligand structure-dependent induced conformational differences of the bound receptor and subsequent interactions with cell specific nuclear cofactors. In this study modeling of NR4A1-piperlongumine interactions showed ligand interactions with multiple amino acid side chains within the LBD (Figure 1). Previous modeling studies showed that NR4A1 binding with structurally diverse ligands exhibited both common and different interactions with amino acid side chain within the LBD. For example, the key amino acid side chain interactions were observed for the following compounds; quercetin (Glu109, Phe112, Leu113, Glu114) [41], 1,1-bis(3′-indolyl)-1-(3,5-dichlorophenyl) methane (Ser110, Glu114, Arg184, Arg232 and Thr236) and resveratrol (Ser110, Leu113, Glu114, Arg184, Thr236, Leu239, and Ile-260) (Zhang et al., 2022). Thus, these structurally-diverse ligands interact with some of the same amino acid side chains but they also exhibit some differences and this could influence their recruitment of other nuclear cofactors and contribute to their activity as selective receptor modulators.

In summary results of this study show that piperlongumine binds the orphan nuclear receptor NR4A1 and acts as an inverse receptor agonist in colon cancer cells. These results indicate that the anticancer activity of piperlongumine is due in part, to its inactivation of NR4A1 and effects of this compound in other disease models where NR4A1 is a drug target are currently being investigated.

## Data Availability

The original contributions presented in the study are included in the article/Supplementary Material, further inquiries can be directed to the corresponding author.

## References

[B1] Adams D. J. Dai M. Pellegrino G. Wagner B. K. Stern A. M. Shamji A. F. (2012). Synthesis, cellular evaluation, and mechanism of action of piperlongumine analogs. Proc. Natl. Acad. Sci. U. S. A. 109 (38), 15115–15120. 10.1073/pnas.1212802109 22949699 PMC3458345

[B2] Alexandre J. R. M. Hu Y. Lu W. Pelicano H. Huang P. (2007). Novel action of paclitaxel against cancer cells: Bystander effect mediated by reactive oxygen species. Cancer Res. 67 (8), 3512–3517. 10.1158/0008-5472.Can-06-3914 17440056

[B3] Burris T. P. Solt L. A. Wang Y. Crumbley C. Banerjee S. Griffett K. (2013). Nuclear receptors and their selective pharmacologic modulators. Pharmacol. Rev. 2, 710–778. 10.1124/pr.112.006833 PMC11060414 23457206

[B4] Castaldo S. A. Freitas J. R. Conchinha N. V. Madureira P. A. (2016). The tumorigenic roles of the cellular REDOX regulatory systems. Oxidative Med. Cell. Longev. 2016, 8413032. 10.1155/2016/8413032 PMC4670861 26682014

[B5] Chatterjee S. Walsh E. N. Yan A. L. Giese K. P. Safe S. Abel T. (2020). Pharmacological activation of Nr4a rescues age-associated memory decline. Neurobiol. Aging 85, 140–144. 10.1016/j.neurobiolaging.2019.10.001 31732218 PMC6917472

[B6] Chen L. Fan F. Wu L. Zhao Y. (2020). The nuclear receptor 4A family members: Mediators in human disease and autophagy. Cell. Mol. Biol. Lett. 25 (1), 48. 10.1186/s11658-020-00241-w 33292165 PMC7640683

[B7] Chio I. I. C. Tuveson D. A. (2017). ROS in cancer: The burning question. Trends Mol. Med. 23 (5), 411–429. 10.1016/j.molmed.2017.03.004 28427863 PMC5462452

[B8] Friesner R. A. Banks J. L. Murphy R. B. Halgren T. A. Klicic J. J. Mainz D. T. (2004). Glide: A new approach for rapid, accurate docking and scoring. 1. Method and assessment of docking accuracy. J. Med. Chem. 47 (7), 1739–1749. 10.1021/jm0306430 15027865

[B9] Friesner R. A. Murphy R. B. Repasky M. P. Frye L. L. Greenwood J. R. Halgren T. A. (2006). Extra precision glide: Docking and scoring incorporating a model of hydrophobic enclosure for protein-ligand complexes. J. Med. Chem. 49 (21), 6177–6196. 10.1021/jm051256o 17034125

[B10] Gabr S. A. Elsaed W. M. Eladl M. A. El-Sherbiny M. Ebrahim H. A. Asseri S. M. (2022). Curcumin modulates oxidative stress, fibrosis, and apoptosis in drug-resistant cancer cell lines. Life 12 (9), 1427. 10.3390/life12091427 36143462 PMC9504331

[B11] Halgren T. A. Murphy R. B. Friesner R. A. Beard H. S. Frye L. L. Pollard W. T. (2004). Glide: A new approach for rapid, accurate docking and scoring. 2. Enrichment factors in database screening. J. Med. Chem. 47 (7), 1750–1759. 10.1021/jm030644s 15027866

[B12] Hammond S. L. Safe S. Tjalkens R. B. (2015). A novel synthetic activator of Nurr1 induces dopaminergic gene expression and protects against 6-hydroxydopamine neurotoxicity *in vitro* . Neurosci. Lett. 607, 83–89. 10.1016/j.neulet.2015.09.015 26383113 PMC4631643

[B13] Hedrick E. Lee S-O. Doddapaneni R. Singh M. Safe S. (2016). NR4A1 antagonists inhibit β1-integrin-dependent breast cancer cell migration. Mol. Cell Biol. 36, 1383–1394. 10.1128/MCB.00912-15 26929200 PMC4836213

[B14] Hedrick E. Lee S-O. Kim G. Abdelrahim M. Jin U-H. Safe S. (2015). Nuclear receptor 4A1 (NR4A1) as a drug target for renal cell adenocarcinoma. PLOS ONE 10 (6), e0128308. 10.1371/journal.pone.0128308 26035713 PMC4452731

[B15] Hu M. Luo Q. Alitongbieke G. Chong S. Xu C. Xie L. (2017). Celastrol-induced Nur77 interaction with TRAF2 alleviates inflammation by promoting mitochondrial ubiquitination and autophagy. Mol. Cell 66 (1), 141–153. 10.1016/j.molcel.2017.03.008 28388439 PMC5761061

[B16] Ito K. Bernardi R. Morotti A. Matsuoka S. Saglio G. Ikeda Y. (2008). PML targeting eradicates quiescent leukaemia-initiating cells. Nature 453 (7198), 1072–1078. 10.1038/nature07016 18469801 PMC2712082

[B17] Jutooru I. Guthrie A. S. Chadalapaka G. Pathi S. Kim K. Burghardt R. (2014). Mechanism of action of phenethylisothiocyanate and other reactive oxygen species-inducing anticancer agents. Mol. Cell Biol. 34 (13), 2382–2395. 10.1128/mcb.01602-13 24732804 PMC4054319

[B18] Karki K. Mohankumar K. Schoeller A. Martin G. Shrestha R. Safe S. (2021). NR4A1 ligands as potent inhibitors of breast cancer cell and tumor growth. Cancers 13 (11), 2682. 10.3390/cancers13112682 34072371 PMC8198788

[B19] Kasiappan R. Jutooru I. Mohankumar K. Karki K. Lacey A. Safe S. (2019). Reactive oxygen species (ROS)-Inducing triterpenoid inhibits rhabdomyosarcoma cell and tumor growth through targeting Sp transcription factors. Mol. Cancer Res. 17 (3), 794–805. 10.1158/1541-7786.Mcr-18-1071 30610105 PMC6397684

[B20] Kim S. J. Kim H. S. Seo Y. R. (2019). Understanding of ROS-inducing strategy in anticancer therapy. Oxidative Med. Cell. Longev. 2019, 5381692. 10.1155/2019/5381692 PMC6939418 31929855

[B21] Kim T. W. Lee S. Y. Kim M. Cheon C. Ko S-G. (2018). Kaempferol induces autophagic cell death via IRE1-JNK-CHOP pathway and inhibition of G9a in gastric cancer cells. Cell Death Dis. 9, 875. 10.1038/s41419-018-0930-1 30158521 PMC6115440

[B22] Kung F-P. Lim Y-P. Chao W-Y. Zhang Y-S. Yu H-I. Tai T-S. (2021). Piperlongumine, a potent anticancer phytotherapeutic, induces cell cycle arrest and apoptosis *in vitro* and *in vivo* through the ROS/akt pathway in human thyroid cancer cells. Cancers 13 (17), 4266. 10.3390/cancers13174266 34503074 PMC8428232

[B23] Kurakula K. Koenis D. S. van Tiel C. M. de Vries C. J. (2014). NR4A nuclear receptors are orphans but not lonesome. Biochim. Biophys. Acta 1843 (11), 2543–2555. 10.1016/j.bbamcr.2014.06.010 24975497

[B24] Lacey A. Hedrick E. Li X. Patel K. Doddapaneni R. Singh M. (2016). Nuclear receptor 4A1 (NR4A1) as a drug target for treating rhabdomyosarcoma (RMS). Oncotarget 7 (21), 31257–31269. 10.18632/oncotarget.9112 27144436 PMC5058754

[B25] Lee S-O. Abdelrahim M. Yoon K. Chintharlapalli S. Papineni S. Kim K. (2010). Inactivation of the orphan nuclear receptor TR3/Nur77 inhibits pancreatic cancer cell and tumor growth. Cancer Res. 70, 6824–6836. 10.1158/0008-5472.CAN-10-1992 20660371 PMC2988472

[B26] Lee S-O. Jin U-H. Kang J. H. Kim S. B. Guthrie A. S. Sreevalsan S. (2014b). The orphan nuclear receptor NR4A1 (Nur77) regulates oxidative and endoplasmic reticulum stress in pancreatic cancer cells. Mol. Cancer Res. 12 (4), 527–538. 10.1158/1541-7786.Mcr-13-0567 24515801 PMC4407472

[B27] Lee S-O. Li X. Hedrick E. Jin U-H. Tjalkens R. B. Backos D. S. (2014a). Diindolylmethane analogs bind NR4A1 and are NR4A1 antagonists in colon cancer cells. Mol. Endocrinol. 28 (10), 1729–1739. 10.1210/me.2014-1102 25099012 PMC4179635

[B28] Liu J-J. Zeng H-N. Zhang L-R. Zhan Y-Y. Chen Y. Wang Y. (2010). A unique pharmacophore for activation of the nuclear orphan receptor nur77 *in vivo* and *in vitro* . Cancer Res. 70 (9), 3628–3637. 10.1158/0008-5472.Can-09-3160 20388790

[B29] Mohankumar K. Lee J. Wu C. S. Sun Y. Safe S. (2018). Bis-indole–derived NR4A1 ligands and metformin exhibit nr4a1-dependent glucose metabolism and uptake in C2C12 cells. Endocrinology 159 (5), 1950–1963. 10.1210/en.2017-03049 29635345 PMC5888234

[B30] Mohankumar K. Li X. Sridharan S. Karki K. Safe S. (2019). Nuclear receptor 4A1 (NR4A1) antagonists induce ROS-dependent inhibition of mTOR signaling in endometrial cancer. Gynecol. Oncol. 154 (1), 218–227. 10.1016/j.ygyno.2019.04.678 31053403 PMC6625344

[B31] Mohankumar K. Li X. Sung N. Cho Y. J. Han S. J. Safe S. (2020). Bis-indole–derived nuclear receptor 4A1 (NR4A1, Nur77) ligands as inhibitors of endometriosis. Endocrinology 161 (4), bqaa027. 10.1210/endocr/bqaa027 32099996 PMC7105386

[B32] O'Hagan H. M. Wang W. Sen S. DeStefano Shields C. Lee S. S. Zhang Y. W. (2011). Oxidative damage targets complexes containing DNA methyltransferases, SIRT1, and polycomb members to promoter CpG islands. Cancer Cell 20 (5), 606–619. 10.1016/j.ccr.2011.09.012 22094255 PMC3220885

[B33] Pan X. Chen G. Hu W. (2022). Piperlongumine increases the sensitivity of bladder cancer to cisplatin by mitochondrial ROS. J. Clin. Laboratory Analysis 36 (6), e24452. 10.1002/jcla.24452 PMC9169161 35466450

[B34] Pearen M. A. Muscat G. E. O. (2010). Minireview: Nuclear hormone receptor 4A signaling: Implications for metabolic disease. Mol. Endocrinol. 24 (10), 1891–1903. 10.1210/me.2010-0015 20392876 PMC5417389

[B35] Pushpakom S. Iorio F. Eyers P. A. Escott K. J. Hopper S. Wells A. (2019). Drug repurposing: Progress, challenges and recommendations. Nat. Rev. Drug Discov. 18 (1), 41–58. 10.1038/nrd.2018.168 30310233

[B36] Ristow M. Zarse K. Oberbach A. Klöting N. Birringer M. Kiehntopf M. (2009). Antioxidants prevent health-promoting effects of physical exercise in humans. Proc. Natl. Acad. Sci. 106 (21), 8665–8670. 10.1073/pnas.0903485106 19433800 PMC2680430

[B37] Safe S. Abbruzzese J. Abdelrahim M. Hedrick E. (2018). Specificity protein transcription factors and cancer: Opportunities for drug development. Cancer Prev. Res. 11 (7), 371–382. 10.1158/1940-6207.Capr-17-0407 29545399

[B38] Safe S. Karki K. (2021). The paradoxical roles of orphan nuclear receptor 4A (NR4A) in cancer. Mol. Cancer Res. 19 (2), 180–191. 10.1158/1541-7786.Mcr-20-0707 33106376 PMC7864866

[B39] Safe S. Shrestha R. Mohankumar K. (2021). Orphan nuclear receptor 4A1 (NR4A1) and novel ligands. Essays Biochem. 65 (6), 877–886. 10.1042/ebc20200164 34096590 PMC11410023

[B40] Schumacker Paul T. (2015). Reactive oxygen species in cancer: A dance with the devil. Cancer Cell 27 (2), 156–157. 10.1016/j.ccell.2015.01.007 25670075

[B41] Shrestha R. Mohankumar K. Jin U. H. Martin G. G. Safe S. (2020). The histone methyltransferase gene G9A is regulated by nuclear receptor 4A1 in alveolar rhabdomyosarcoma cells. Mol. Cancer Ther. 20 (3), 612–622. 10.1158/1535-7163.MCT-20-0474 33277444 PMC7933077

[B42] Shrestha R. Mohankumar K. Martin G. Hailemariam A. Lee S-O. Jin U-H. (2021). Flavonoids kaempferol and quercetin are nuclear receptor 4A1 (NR4A1, Nur77) ligands and inhibit rhabdomyosarcoma cell and tumor growth. J. Exp. Clin. Cancer Res. 40 (1), 392. 10.1186/s13046-021-02199-9 34906197 PMC8670039

[B43] Simůnek T. Stérba M. Popelová O. Adamcová M. Hrdina R. Gersl V. (2009). Anthracycline-induced cardiotoxicity: Overview of studies examining the roles of oxidative stress and free cellular iron. Pharmacol. Rep. 61 (1), 154–171. 10.1016/s1734-1140(09)70018-0 19307704

[B44] Sosa V. Moliné T. Somoza R. Paciucci R. Kondoh H. Lleonart M. E. (2013). Oxidative stress and cancer: An overview. Ageing Res. Rev. 12 (1), 376–390. 10.1016/j.arr.2012.10.004 23123177

[B45] Sritharan S. Sivalingam N. (2021). A comprehensive review on time-tested anticancer drug doxorubicin. Life Sci. 278, 119527. 10.1016/j.lfs.2021.119527 33887349

[B46] Trachootham D. Alexandre J. Huang P. (2009). Targeting cancer cells by ROS-mediated mechanisms: A radical therapeutic approach? Nat. Rev. Drug Discov. 8 (7), 579–591. 10.1038/nrd2803 19478820

[B47] Yoshida T. Goto S. Kawakatsu M. Urata Y. Li T-S. (2012). Mitochondrial dysfunction, a probable cause of persistent oxidative stress after exposure to ionizing radiation. Free Radic. Res. 46 (2), 147–153. 10.3109/10715762.2011.645207 22126415

[B48] Zhan Y. Y. Chen Y. Zhang Q. Zhuang J. J. Tian M. Chen H. Z. (2012). The orphan nuclear receptor Nur77 regulates LKB1 localization and activates AMPK. Nat. Chem. Biol. 8 (11), 897–904. 10.1038/nchembio.1069 22983157

[B49] Zhang L. Martin G. Mohankumar K. Hampton J. T. Liu W. R. Safe S. (2022). Resveratrol binds nuclear receptor 4A1 (NR4A1) and acts as an NR4A1 antagonist in lung cancer cells. Mol. Pharmacol. 102 (2), 80–91. 10.1124/molpharm.121.000481 35680166 PMC9341251

[B50] Zhang L. Wang Q. Liu W. Liu F. Ji A. Li Y. (2018). The orphan nuclear receptor 4A1: A potential new therapeutic target for metabolic diseases. J. Diabetes Res. 2018, 9363461. 10.1155/2018/9363461 30013988 PMC6022324

[B51] Zhao Z. Wang Y. Gong Y. Wang X. Zhang L. Zhao H. (2022). Celastrol elicits antitumor effects by inhibiting the STAT3 pathway through ROS accumulation in non-small cell lung cancer. J. Transl. Med. 20 (1), 525. 10.1186/s12967-022-03741-9 36371217 PMC9652895

[B52] Zhu J. Song X. Lin H-P. Young D. C. Yan S. Marquez V. E. (2002). Using cyclooxygenase-2 inhibitors as molecular platforms to develop a new class of apoptosis-inducing agents. JNCI J. Natl. Cancer Inst. 94 (23), 1745–1757. 10.1093/jnci/94.23.1745 12464646

