# Supplementary figures and images for: Piperlongumine is a ligand for the orphan nuclear receptor 4A1 (NR4A1)
Source: Front Pharmacol. 2023 Sep 21;14:1223153. doi: 10.3389/fphar.2023.1223153 (PMC10551445; doi:10.3389/fphar.2023.1223153)

Supplemental Figure 1

A.

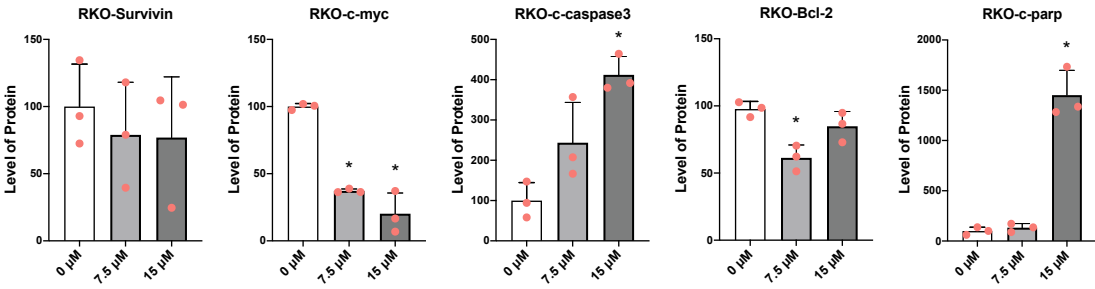

B.

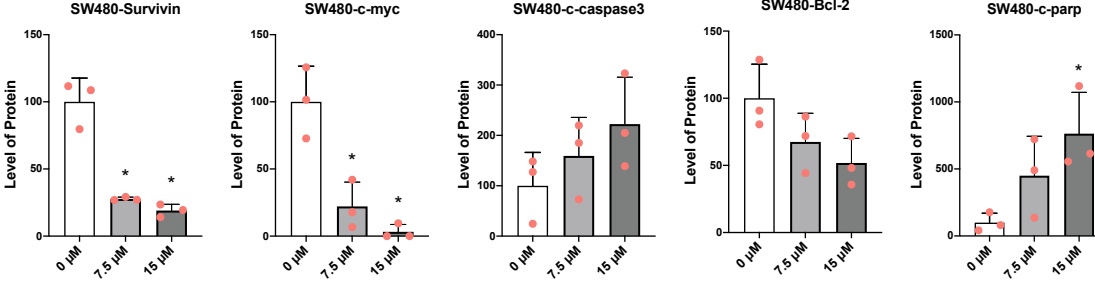

C.

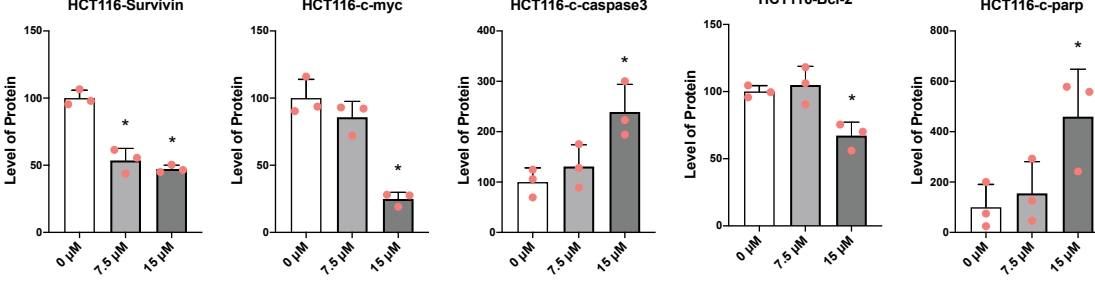

D. Piperlongumine:  $\beta$ 1-Intergrin Expression

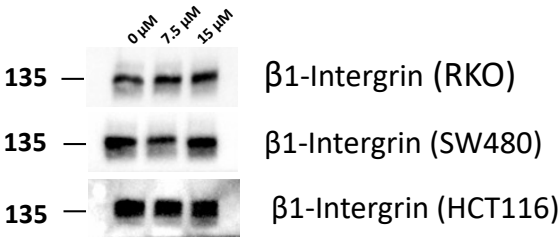

E.

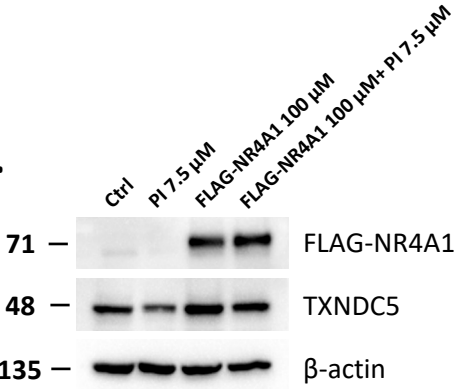

Supplement: Supplementary file 1 [file DataSheet2.PDF]
