# Supplementary material for: Piperlongumine is a ligand for the orphan nuclear receptor 4A1 (NR4A1)
Source: Front Pharmacol. 2023 Sep 21;14:1223153. doi: 10.3389/fphar.2023.1223153 (PMC10551445; doi:10.3389/fphar.2023.1223153)
Supplement: Supplementary file 2 [file DataSheet1.DOCX]

Supplementary Material

PIPERLONGUMINE IS A LIGAND FOR THE ORPHAN NUCLEAR RECEPTOR 4A1 (NR4A1)

Lei Zhang1, Greg Martin1, Kumaravel Mohankumar1, Gus A. Wright2, Fuada Mariyam1, and Stephen Safe1*

*** Correspondence:** Stephen Safe: ssafe@cvm.tamu.edu

# Supplementary Figures and Tables

## Supplementary Figures

**
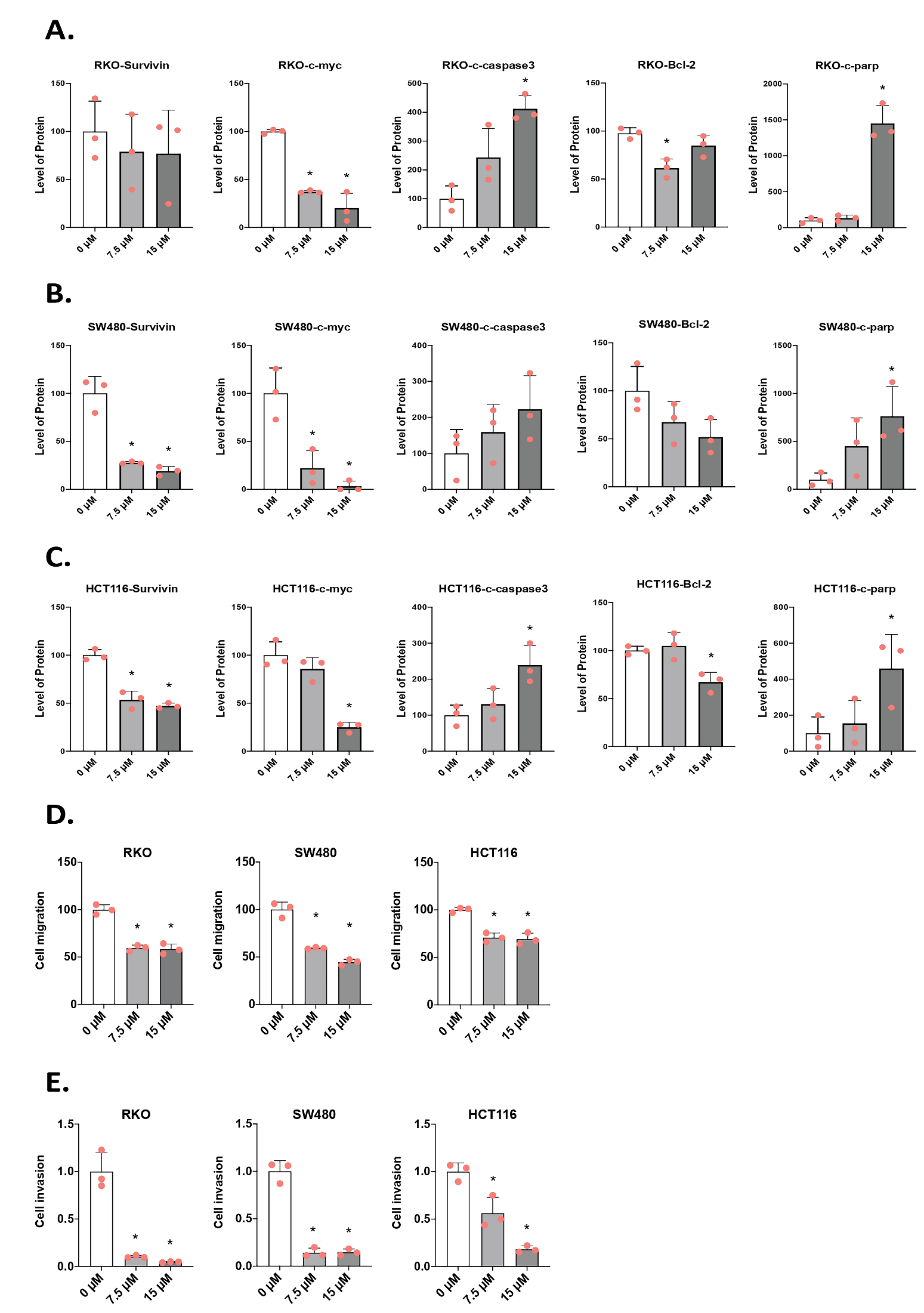
**

**Supplementary Figure 1.** Quantitation of western blots and migration/invasion.

Cells were treated as indicated and western blots of whole cell lysates were quantitated relative to β-actin with controls set at 100% from data in A) Figure 3A, B) Figure 3B, C) Figure 3C. Quantitation of migration D) Figure 3D and invasion E) Figure 3E results were determined as outlined in the Methods.

## Supplementary Table

| Name of antibody | Vendor | Catlog # | Western blotting (Dilution) | ChIP (Mass) |
| --- | --- | --- | --- | --- |
| β-actin | Sigma | A5316 | 3000 |  |
| NR4A1 | Abcam | 109180 | 1000 | 3µg |
| Survivin | R&D | AF886 | 500 |  |
| c-Myc | Abcam | Ab185656 | 500 |  |
| c-Parp | Cell Signaling | 9541 | 1000 |  |
| c-Caspase 3 | Invitrogen | PA5-114687 | 500 |  |
| Bcl-2 | Cell Signaling | 4223 | 500 |  |
| txndc5 | GeneTex | GTX106914 | 1000 |  |
| sestrin2 | Abcam | 18907 | 1000 |  |
| ampk-α | Cell Signaling | 5831 | 500 |  |
| p-ampk-α | Cell Signaling | 2535 | 500 |  |
| idh1 | Cell Signaling | 8137 | 500 |  |
| m-TOR | Cell Signaling | 2972 | 500 |  |
| p-m-TOR | Abcam | 109268 | 500 |  |
| p70S6 | Cell Signaling | 9202 | 500 |  |
| p-p70s6 | Cell Signaling | 9205 | 500 |  |
| 4e-bp1 | Cell Signaling | 9644 | 1000 |  |
| p-4e-bp1 | Cell Signaling | 9451 | 1000 |  |
| G9a | Cell Signaling | 68851 | 1000 |  |
| Sp1 | Abcam | 13370 | 1000 | 3µg |
| pol ii | Abcam | 264350 |  | 3µg |
| Igg | Abcam | 171870 |  | 3µg |

**Supplementary Table 1.**  Sources of antibodies used in this study.
